# Feasibility of a Multifaceted Social Emergency Medicine Curriculum for Emergency Medicine Residents

**DOI:** 10.5811/westjem.59009

**Published:** 2023-05-05

**Authors:** Erin F. Shufflebarger, Melissa Willett, Sylvia Y. Sontheimer, Sherell Hicks, Charles A. Khoury, Lauren A. Walter

**Affiliations:** * University of Alabama at Birmingham Heersink School of Medicine, Department of Emergency Medicine, Birmingham, Alabama; † Boston Medical Center, Department of Emergency Medicine, Boston, Massachusetts

## Abstract

**Introduction:**

Emergency physicians are in a unique position to impact both individual and population health needs. Despite this, emergency medicine (EM) residency training lacks formalized education n the social determinants of health (SDoH) and integration of patient social risk and need, which are core components of social EM (SEM). The need for such a SEM-based residency curriculum has been previously recognized; however, there is a gap in the literature related to demonstration and feasibility. In this study we sought to address this need by implementing and evaluating a replicable, multifaceted introductory SEM curriculum for EM residents. This curriculum is designed to increase general awareness related to SEM and to increase ability to identify and intervene upon SDoH in clinical practice.

**Methods:**

A taskforce of EM clinician-educators with expertise in SEM developed a 4.5-hour educational curriculum for use during a single, half-day didactic session for EM residents. The curriculum consisted of asynchronous learning via a podcast, four SEM subtopic lecture didactics, guest speakers from the emergency department (ED) social work team and a community outreach partner, and a poverty simulation with interdisciplinary debrief. We obtained pre- and post- intervention surveys.

**Results:**

A total of 35 residents and faculty attended the conference day, with 18 participants completing the immediate post-conference survey and 10 participants completing the two-month delayed, post-conference survey. Post-survey results demonstrated improved awareness of SEM concepts and increased confidence in participants’ knowledge of community resources and ability to connect patients to these resources following the curricular intervention (25% pre-conference to 83% post-conference). In addition, post-survey assessment demonstrated significantly heightened awareness and clinical consideration of SDoH among participants (31% pre-conference to 78% post-conference) and increased comfort in identifying social risk in the ED (75% pre-conference to 94% post-conference). Overall, all components of the curriculum were evaluated as meaningful and specifically beneficial for EM training. The ED care coordination, poverty simulation, and the subtopic lectures were rated most meaningful.

**Conclusion:**

This pilot curricular integration study demonstrates feasibility and the perceived participant value of incorporating a social EM curriculum into EM residency training.

## BACKGROUND

The importance of addressing social determinants of health (SDoH) as a part of patient care is widely recognized. The World Health Organization’s Commission on SDoH emphasized the importance of increased awareness as well as education and training specifically related to SDoH as a way to improve health equity.^1^ There is growing interest in incorporating SDoH into the undergraduate medical education curriculum, although this education is not standardized and is not yet available to every medical student.^2^ Within graduate medical education, the emphasis on SDoH education has predominantly been within primary care specialties (ie, internal medicine, family medicine, and pediatrics) due to the longitudinal patient relationships typically present in these specialties. However, primary care residency training programs still lack uniform and standardized SDoH curriculum content, implementation, and evaluation.^3^

Although emergency medicine (EM) is not considered a primary care specialty, emergency physicians are routinely confronted with SDoH, social needs, and the reality of health disparities. The emergency department (ED) has been described as “the social barometer of its community.”^4^ Given the unique relationship between SDoH and acute care in the ED, the field of social emergency medicine (SEM) has emerged, in which both individual and population health needs are considered.^5^ Research in this field has led to the implementation of many effective ED-based interventions to address population health needs in domains including access to care, exposure to violence/crime, language/literacy/healthcare literacy, and poverty.^6^

Despite this recognized overlap between SDoH and EM, medical training, specifically EM residency training, often lacks a formalized curriculum related to SEM. A need has now developed for training in SDoH and application of this knowledge to practice.^4^ Existing literature demonstrates the feasibility of integrating SDoH-specific education as a part of an EM clerkship.^7^ The need for a SEM curriculum adapted specifically for EM residency training has been described and called for in previous literature,^8^ and objectives for such a curricular addition have been proposed.^9^ However, there is a gap in the literature related to the feasibility of such a curriculum addition. In this study we sought to address this need as we evaluated the feasibility of a multifaceted, immersive, introductory SEM curriculum for use in EM resident education.

## OBJECTIVES

### Our study goals were as follows

1. To design, implement, and evaluate the feasibility of a replicable, multifaceted SEM curriculum for EM residents.
2. To increase EM residents’ level of awareness related to SEM and to improve their ability to identify and intervene in SDoH in clinical practice.

## CURRICULAR DESIGN

### Study Design and Protocol

We developed this curriculum using the six-step approach for curriculum development by Kern et al.^10^ The overall need for a SEM curriculum was established in the literature as previously discussed and was confirmed in a needs assessment conducted among EM residents. Next, following Kern’s framework, we established goals, objectives, and educational strategies to meet these objectives. The curriculum was then implemented and subsequently evaluated by the learners.^10^

A task force comprised of EM clinician-educators, including a SEM fellowship director and fellow, an EM residency program director, and an EM resident and senior medical student with specific interest in SEM, was assembled at the University of Alabama at Birmingham (UAB). The pilot “SEM curriculum” was designed as a single didactic and experiential learning block. It included four continuous hours of resident education time plus 30 minutes of asynchronous pre-learning with debrief, for a total of 4.5 hours of didactic time. This study was reviewed and subsequently determined to be exempt by the UAB Institutional Review Board.

The final curriculum (Table 1) included asynchronous flipped learning via a podcast,^11^ four subtopic lecture didactics, guest speakers from the ED social work team and a community representative, and a poverty simulation and debrief.^12^, ^13^ The material for the subtopic lectures was chosen considering the patient population frequently encountered by the resident learners and, when replicated, can be adjusted to meet the needs of the learners and their surrounding community. The curriculum was delivered by members of the curriculum development task force along with simulation faculty in April 2021 via videoconferencing due to COVID-19 restrictions.

### Study Setting and Population

The UAB Emergency Medicine Residency Program is a three-year ACGME-accredited residency program in Birmingham, Alabama with 32 residents as of July 2020. The program is accredited by the Accreditation Council for Graduate Medical Education. Residents are allotted protected time from clinical duties to attend weekly didactics for a 4–5 hour block.

### Key Outcome Measures

We developed two participant surveys, including a “pre-intervention” and “post-intervention” survey, and distributed the survey by email to UAB EM residents and participating faculty to evaluate the effect and impact of the virtual curriculum as well as generate general feedback. Survey responses were kept anonymous, but pre- and post-intervention surveys were matched using a unique identifier. Surveys included general demographic information (eg, gender, race) and subjective information measured by a Likert scale including self-perceived attitude and comfort level regarding identifying and addressing SDoH in the ED setting. The pre-conference survey also incorporated the “Medical Condition Regard Scale” (MCRS) to assess participants’ general attitude toward patients with social needs. The MCRS has prior evidence of validity in a similar population and measures “the degree to which the respondents find patients with a given medical condition enjoyable, treatable, and worthy of medical resources.”^14^ The surveys focused on the Kirkpatrick Model of Evaluation levels 1 and 2, evaluating learner reaction to and satisfaction with the curriculum as well as measuring learner attitude change as a result of the curriculum.^15^

### Data Analysis

We used JotForm (Jotform, Inc, San Francisco, CA) to create the survey and collect all survey data. Descriptive statistics were conducted using frequencies and percentages for categorical data. We performed paired sample *t*-test analysis to assess whether there was a difference between matched pre- and post- survey responses from residents and other participants. *P*-values <0.05 were considered to be statistically significant. We performed all statistical analyses using JMP Pro 14 (JMP Statistical Discovery, LLC, Cary, NC).^16^

## IMPACT/EFFECTIVENESS

### Results

A total of 23 residents (71.9%) along with 12 other participants including EM faculty and a medical student attended the conference day. Eighteen people (51.4% of total participants) including 14 residents (60.9% of participating residents) completed the immediate post-conference survey, and 10 people (28.6% of total participants) including seven residents (30.4% of participating residents) completed the two-month delayed, post-conference survey.

Participant pre- and immediate post-survey results are displayed in Table 2. Before the conference, only 31.3% of responding participants reported prior training on identifying and intervening on SDoH. After the conference, participants were significantly more likely to report being aware of and familiar with local community resources to address SDoH and were also more confident in their knowledge of these community resources and their ability to connect patients to them. In addition, the post-conference data indicated that participants were significantly more likely to consider SDoH when providing treatment to ED patients and were significantly more comfortable with identifying social risk in the ED.

A majority of the participants reported caring for greater than 15 patients with social needs in the ED in the previous month, with 44% of the participants reporting caring for greater than 30 patients with social needs. The most commonly encountered or anticipated barriers to addressing SDoH in the ED setting were thought to be emergency physician (EP) time constraints, lack of knowledge of resources, and availability of resources.

As demonstrated in Table 3, respondents reported overall positive attitude toward patients experiencing social needs (eg, homelessness, food insecurity). However, a majority of participants (59%) disagreed with the statement that they enjoy giving extra time to patients like this. As resident postgraduate (PGY) year increased, respondents became more likely to disagree with the following statement: “I feel especially compassionate toward patients like this,” with zero percent of PGY-1 participants, 27% of PGY-2 participants, and 57% of PGY-3 participants disagreeing with this statement. The MCRS survey was repeated in the two-month delayed, post-conference survey. Unfortunately, only four participants could be matched to their pre-survey responses; therefore, we did not analyze this data for trends.

Feedback received following completion of the course was positive. Seventeen of eighteen (94.4%) of the respondents reported an improved understanding of the topic. Sixteen of eighteen (88.9%) respondents would recommend this curriculum to other EM residents. Similarly, 83% of respondents reported that this training increased their confidence in caring for patients with social needs. Overall, all components of the curriculum were felt to be beneficial and meaningful to the training. The ED care coordination, poverty simulation, and the subtopic lectures were rated most meaningful (Appendix 1).

## DISCUSSION

Emergency physicians encounter patients with both acute and chronic medical and social needs on a daily basis. Just as we expect every practicing EP to be trained and ready to appropriately respond to a patient presenting with stroke symptoms, we should also expect every EP to be trained and ready to appropriately respond to a patient presenting with social need. This requires appropriate education and training. There is exciting work being done in the realm of education related to SDoH in EM. The feasibility of integrating SDoH-specific education into undergraduate medical education was described in 2019 when a three-part curriculum was integrated into an EM clerkship.^7^ The concepts of SEM have also recently been incorporated into resident education at one institution using simulation with eight cases focusing on health equity.^17^ Despite these recent advances, a formalized, standardized residency training on SDoH and SEM is missing from most required curricula. Less than one-third of our participants reported receiving previous training on how to identify and intervene on SDoH.

This study demonstrates that the implementation of an introductory virtual SEM curriculum for EM residents is feasible and effective. Given the multifaceted approach, we anticipate that other institutions may be able to use or incorporate some or all of this framework, modifying it to fit the needs of their learners and local SDoH. The curriculum is intended to be locally relevant but can be easily replicated using the same model. Some components of the curriculum can be used directly (asynchronous podcast and poverty simulation), while other didactic components should be tailored to the specific needs of the local community (Table 1).

Ideally, EM training programs will be able to implement a longitudinal, integrated, SDoH-focused curriculum to better equip EPs to care for the social needs of their patients.^9^ However, this half-day curriculum serves as a demonstration of a focused didactic block that can be used either as an introduction to a longitudinal curriculum or as the first step in integrating SEM education into the resident curriculum. While this initial curriculum took only four hours of allotted resident conference time, participant surveys indicate that implementation of a single conference day was effective. We anticipate a longitudinal SEM curriculum would be just as effective and comprehensive, if not more so.

In the specialty of EM, burnout rates are high, and successful mechanisms to reduce burnout are needed.^18^ One component of burnout is emotional erosion, or “the transition of enthusiasm and compassion at the beginning of practice to anger, cynicism, and bitterness.”^19^ An interesting finding of the pre-survey MCRS was that participants’ feelings of compassion toward patients with social needs decreased with each year of residency training. While the significance of this should be interpreted with caution as the sample size was small, this trend warrants further consideration.

Axelson et al. proposed that an under-recognized contributor to burnout is a sense of futility in the daily practice of EM due to lack of training to identify and intervene on SDoH.^8^ This makes sense, as it could be frustrating to consistently be confronted with an issue that you have not been adequately trained to address. It is reasonable to consider that increasing SDoH education for EPs could be a useful tool for reducing burnout in the specialty. Perhaps including this education early in residency, even as early as during intern orientation, could mitigate this contributor to burnout if EM trainees felt more equipped to provide this compassionate, effective care and address patients’ social needs from the start of training. The impact of SDoH education on markers of EP burnout is an important factor to consider with future educational interventions.

## LIMITATIONS

This was a single-center, pilot study involving one EM residency program and, therefore, participant numbers were small. Further implementation at other sites as a multicenter study will be necessary to further investigate the generalizability of the results of this pilot study to all EM residency programs. Additionally, this curriculum was implemented during the COVID-19 pandemic. For the safety of all participants and guest speakers, the entire curriculum including the simulation took place virtually using online video conferencing. This virtual learning platform introduces limitations including technical difficulties and reduced learner engagement.^20^ The response rate fell with each subsequent survey despite multiple email reminders to complete the surveys, increasing the possibility of nonresponse bias.

Future in-person course delivery should attempt to increase immediate post-survey response rates by offering participants a variety of options for survey completion (eg, web-based survey, written survey). We were also unable to supplement the classroom and simulation experience with an in-person community experience (eg, service activity, touring community resources) given these restrictions. When planning future curriculum innovation, we will seek to expand this SEM resident curriculum with the addition of a community engagement component.

## CONCLUSION

Emergency physicians are in a unique position to impact both individual as well as community and population health. Despite this, formalized resident training in the social determinants of health is lacking. This single pilot study demonstrates the feasibility and perceived participant value of incorporating a social emergency medicine curriculum into residency training.

## Supplementary Information



## Figures and Tables

**Table 1:** Components of a social emergency medicine curriculum.

Component	Description	Time allotted	To replicate
1. Pre-didactic asynchronous learning	Announce Podcast “Episode 4 – Social Determinants of Health and Unmet Needs in the Emergency Department”^11^	30 minutes	See Reference 11 for podcast
2. Subtopic lectures	PowerPoint slide presentations Intro to SEM/Asynchronous Debrief Incarceration Firearm Violence Homelessness	60 minutes (10–15 minutes each)	Tailor topics to local community need. Specific materials used here can be provided upon request to corresponding author
3. Guest speaker from community resource	The executive director of a local homeless shelter spoke about the many resources provided by this shelter, as well as about the population that the shelter serves and the interaction between this population and the medical community.	30 minutes	Contact local community partner to present
4. ED care coordination presentation	Members from the ED Care Coordination and Social Services team spoke about available resources for ED patients and how clinicians can connect patients with these resources.	30 minutes	Contact ED social services to present
5. Poverty simulation	Led by the UAB Office of Interprofessional Simulation, the “Poverty Simulation” is an interactive experience “designed to raise awareness of the challenges that individuals may face when living in low-income situations.”^12^ While this simulation is typically an in-person event, given COVID-19 restrictions an online interactive simulation, SPENT, was used and the interprofessional debriefing took place by video conferencing.^13^	2 hours	See Reference 13 for virtual poverty simulation

*SEM*, social emergency medicine; *ED*, emergency department; *UAB*, University of Alabama at Birmingham; *COVID-19*, coronavirus disease 2019.

**Table 2:** Survey results, [n (%)].

Survey question	Pre-survey response (n=32)	Post-survey response (n=18)
The emergency department (ED) is an appropriate venue to connect patients with community resources.		
Strongly agree/Agree	30 (93.8)	17 (94.4)
Strongly disagree/Disagree	2 (6.3)	1 (5.6)
I feel comfortable identifying social need (ex: homelessness, food insecurity) in the ED.		
Strongly agree/Agree	28 (87.5)	17 (94.5)
Strongly disagree/Disagree	4 (12.5)	1 (5.6)
I feel comfortable identifying social risk (ex: risk of worse health outcome for certain races) in the ED.*		
Strongly agree/Agree	24 (75.0)	17 (94.5)
Strongly disagree/Disagree	8 (25.0)	1 (5.6)
I have been trained to identify and intervene on social determinants of health (SDoH).*		
Strongly agree/Agree	10 (31.3)	14 (77.8)
Strongly disagree/Disagree	22 (68.8)	4 (22.2)
I am aware of and familiar with local community resources to address social determinants of health.*		
Strongly agree/Agree	18 (56.3)	16 (88.9)
Strongly disagree/Disagree	14 (43.8)	2 (11.1)
I feel confident in my knowledge about community resources and ability to connect patients to them.*		
Strongly agree/Agree	8 (25.0)	15 (83.3)
Strongly disagree/Disagree	24 (75.0)	3 (16.7)
I frequently encounter patients in the ED with social need that impacts their health.		
Strongly agree/Agree	31 (96.9)	18 (100.0)
Strongly disagree/Disagree	1 (3.1)	0 (0.0)
I frequently encounter patients in the ED with social risk that impacts their health.		
Strongly agree/Agree	31 (96.9)	18 (100.0)
Strongly disagree/Disagree	1 (3.1)	0 (0.0)
I frequently consider SDoH when providing treatment for my patients in the ED.		
Strongly agree/Agree	21 (65.6)	17 (94.5)
Strongly disagree/Disagree	11 (34.4)	1 (5.6)

* Paired samples, P<.05

*ED*, emergency department; *SDoH*, social determinants of health.

**Table 3:** MCRS* survey results, stratified by training year [n(%)].

Survey question	Pre-survey response				

	Total (n=32)	PGY-1 (n=7)	PGY-2 (n=11)	PGY-3 (n=7)	Attending (n=5)
I prefer not to work with patients like this.					
Agree	4 (12.5)	1 (14.3)	2 (18.2)	0 (0.0)	1 (20.0)
Disagree	28 (87.5)	6 (85.7)	9 (81.8)	7 (100.0)	4 (80.0)
Patients like this irritate me.					
Agree	4 (12.5)	1 (14.3)	3 (27.3)	0 (0.0)	0 (0.0)
Disagree	28 (87.5)	6 (85.7)	8 (72.7)	7 (100.0)	5 (100.0)
I enjoy giving extra time to patients like this.					
Agree	13 (40.6)	3 (42.9)	5 (45.5)	1 (14.3)	2 (40.0)
Disagree	19 (59.4)	4 (57.1)	6 (54.6)	6 (85.7)	3 (60.0)
Patients like this are particularly difficult for me to work with.					
Agree	10 (31.3)	2 (28.6)	5 (45.5)	1 (14.3)	1 (20.0)
Disagree	22 (68.8)	5 (71.4)	6 (54.6)	6 (85.7)	4 (80.0)
Working with patients like this is satisfying.					
Agree	20 (62.5)	5 (71.4)	7 (63.6)	3 (42.9)	3 (60.0)
Disagree	12 (37.5)	2 (28.6)	4 (36.4)	4 (57.1)	2 (40.0)
I feel especially compassionate toward patients like this.					
Agree	23 (71.9)	7 (100.0)	8 (72.7)	3 (42.9)	3 (60.0)
Disagree	9 (28.1)	0 (0.0)	3 (27.3)	4 (57.1)	2 (40.0)
I can usually find something that helps patients like this feel better.					
Agree	20 (62.5)	5 (71.4)	7 (63.6)	5 (71.4)	1 (20.0)
Disagree	12 (37.5)	2 (28.6)	4 (36.4)	2 (28.6)	4 (80.0)
There is little I can do to help patients like this.					
Agree	15 (46.9)	3 (42.9)	5 (45.5)	3 (42.9)	3 (60.0)
Disagree	17 (53.1)	4 (57.1)	6 (54.6)	4 (57.1)	2 (40.0)
Treating patients like this is a waste of medical dollars.			2 (18.2)		
Agree	3 (9.4)	1 (14.3)	9 (81.8)	0 (0.0)	0 (0.0)
Disagree	29 (90.6)	6 (85.7)		7 (100.0)	5 (100.0)

* *MCRS*, Medical Condition Regard Scale; PGY, postgraduate year.
