# Supplementary material for: Feasibility of a Multifaceted Social Emergency Medicine Curriculum for Emergency Medicine Residents
Source: West J Emerg Med. 2023 May 5;24(3):495–501. doi: 10.5811/westjem.59009 (PMC10284534; doi:10.5811/westjem.59009)
Supplement: Supplementary file 1 [file wjem-24-495-s001.docx]

Appendix 1. SEM Curriculum Component Evaluation [n(%)]

|  | To what extent did each of the following components add meaningfully to your training? | | | |
| --- | --- | --- | --- | --- |
| Component | Not at all | Slightly | Moderately | Significantly |
| Pre-didactic asynchronous learning | 1 (5.6) | 3 (16.7) | 9 (50.0) | 5 (27.8) |
| Subtopic Lectures | 1 (5.6) | 1 (5.6) | 7 (38.9) | 9 (50.0) |
| Guest speaker from community resource | 1 (5.6) | 4 (23.5) | 5 (29.4) | 7 (41.2) |
| ED care coordination presentation | 0 (0.00) | 2 (11.8) | 5 (29.4) | 10 (58.8) |
| Poverty simulation | 2 (11.8) | 3 (17.7) | 3 (17.7) | 9 (52.9) |
